# Associations of Work-Related Injuries and Stress to Family and Youth Wellbeing among U.S. Latino/a Immigrant Cattle Feedyard Workers

**DOI:** 10.3390/ijerph20043361

**Published:** 2023-02-14

**Authors:** Gustavo Carlo, Meredith McGinley, Sahitya Maiya, Athena K. Ramos

**Affiliations:** 1 School of Education, University of California, Irvine, CA 92697, USA; 2 Department of Psychology, University of Wisconsin, Parkside, Kenosha, WI 53144, USA; 3 Department of Human Development and Family Studies, University of New Hampshire, Durham, NH 03824, USA; 4 College of Public Health, University of Nebraska Medical Center, Omaha, NE 68198, USA

**Keywords:** agricultural workers, Latino/Hispanic, occupational safety, mental health, prosocial behaviors

## Abstract

**Simple Summary:**

Based on primarily on cultural models of stress and the limited prior research, we examined whether work-related stress and lifetime injuries in cattle feedyard workers would be linked to more depressive symptoms and less life satisfaction, as well as to more family conflict and less youth wellbeing. Generally, as expected, we found that work-related stress and lifetime injuries were predictive of their depression, which in turn, predicted more family conflict and less youth helping behaviors. These findings are the first to demonstrate the deleterious possible consequences of work-related stress and injuries in U.S. Latino immigrant cattle feedyard workers to their families and youth. Such findings suggest the need for greater attention to stress and injuries in the workplace and their possible impact on family functioning and point to the development of intervention efforts aimed at reducing such stress, increasing access to mental health services, and developing strong worker safety training programs.

**Abstract:**

Based on the Ecological Stress-Based Model of Immigrant Worker Safety and Health, we hypothesized that occupational stress and physical safety would be negatively linked to workers’ depression, which in turn, would increase family conflict and decrease youth prosocial behaviors. A total of 242 Latino immigrant cattle feedyard workers from Nebraska and Kansas (90.9% male; *M* age = 37.7 years) answered questions assessing depression, occupational stress, whether they had ever been injured at work, familial conflict, and youth prosocial behaviors. All four indirect relations among occupational stress and injury and the outcomes (family conflict and youth prosocial behaviors) via depressive symptomatology were significant. Additionally, ever injured was negatively related to youth prosocial behaviors and occupational stress was positively related to youth prosocial behaviors. The findings support our model and suggest that increased stress and work-related injuries on cattle feedyards are linked to mental health problems, which in turn, is linked to more conflict experienced at home and less youth prosocial behaviors. Feedyard employers should focus on improving safety culture including providing robust training in the workplace. Practical implications to improve availability and access to mental and behavioral health resources to mitigate negative family outcomes are provided.

## 1. Introduction

### 1.1. Cattle Feedyards and Cattle Feedyard Workers in the United States

Cattle feedyard workers in the United States are at high risk for injuries and health problems [1]. Cattle production is considered one of the most important and economically productive agricultural sectors, but agricultural workers, including cattle feedyard workers, display disproportionately high rates of injuries, with 4.9 injuries per 100 full-time equivalents (FTEs) in agriculture and in beef cattle ranching (including feedlots) compared to 2.9 injuries per 100 FTEs across all industries [2]. Given these numbers and the stressful conditions under which cattle feedyard establishments operate, one might expect that there may be impacts to workers and their families outside of the work environment. However, there is a dearth of work that examines the possible consequences of worker injuries and stress on family functioning and youth social development. Moreover, work on possible intervening mechanisms in the relations between workers’ injuries and stress and their family functioning and youth development is also absent.

A cattle feedyard is the setting of the final stage of production where cattle spend approximately 3–6 months as they are fed to market weight [3]. The cattle feeding industry in the U.S. is concentrated in the Great Plains [4]. States such as Nebraska and Kansas are some of the most productive beef cattle states, having respectively the second and third highest numbers of cattle on feed in the country following Texas [5]. Large operations, those with capacities over 32,000 head of cattle, market about 40% of all U.S. cattle [4], but also are associated with increased injury risk among workers [6].

In 2018, there were 14,479 cattle feedyard workers in the U.S., with over 41% of workers employed in Nebraska and Kansas [1]. It is estimated that approximately half of the cattle feedyard workforce consists of immigrants, hailing primarily from Latin America [7]. Immigrant workers are considered a “vulnerable” worker population by the National Institute for Occupational Safety and Health (NIOSH). Many of these Latino immigrant workers may not have had previous experience working with large livestock, and those with limited English proficiency may be less likely to receive job-related training [8]. Immigrant workers may face language, cultural, and contextual challenges (e.g., legal status, mobility, and job security) [9,10]. Many immigrant workers work for low wages and live in poverty by U.S. standards to support their family, whom may or may not be accompanying them. Immigrant workers often experience discrimination and may not understand their labor rights in the U.S. [11,12]. Many workers, particularly those who have various employer-based visas and those who are undocumented, are also fearful to speak up about hazardous conditions or unfair treatment. Stressors among Latino immigrants such as those associated with discrimination and immigration-related fear may increase the risk for poor mental health [13,14]. Outside of work, many of the rural Midwestern communities in Nebraska and Kansas where immigrant agricultural workers live have limited experience with integrating immigrant newcomers [8], are geographically isolated, and have limited access to healthcare services [14,15].

Those challenges place additional demands on cattle feedyard workers and their families, especially those residing and working in the Midwestern part of the U.S. Scholars have posited work-family conflict models that conceptually outline how work- and family-related characteristics (e.g., stress, demands) can ultimately undermine farmworkers’ health and wellbeing [14]. Indeed, there is substantive evidence that negative work-related characteristics experienced by farmworkers (e.g., work-related stress, lack of work flexibility, hazardous working conditions) are linked to workers’ mental health problems [12,14,16,17]. However, such evidence in Latino cattle feedyard workers is limited. For example, need for recovery, a work-related emotional state that indicates excessive effort and predictive of physical and mental fatigue, has been associated with higher job demands and lower decision latitude among cattle feedyard workers [18]. In another study, occupational stress (i.e., job-related demands) was positively predictive of workers’ depression (i.e., negative mood disorder), anxiety, and need for recovery but negatively associated with life satisfaction [19].

### 1.2. Ecological-Stress Based Model of Immigrant Worker Safety and Health

Despite these recent advances in understanding outcomes associated with cattle feedyard workers’ health and injuries, attention to possible effects of cattle feedyard worker injuries and stress on their family functioning and youth developmental outcomes is virtually nonexistent. The Ecological Stress-Based Model of Immigrant Worker Safety and Health [19] outlines historical- and contextual-related stressors (e.g., work-related, major life stressors), as well as, intrapersonal (e.g., coping, mental health) factors that are posited to impact workers’ health outcomes. Building on this model, workers’ stress and injuries are hypothesized to have spillover or cascading effects on family functioning and their youth development. These expectations follow logically from stress and coping models [20], family stress models [21], and ecocultural stress-based models [22,23]. Stress and coping models, for example, suggest that exposure to stress and injuries can deplete resources [24], which can make persons prone to experience depressive symptoms and lower life satisfaction. That is, work-related injuries and stress might result in less psychological energy and undermine positive mood and sense of satisfaction. There is a substantive body of work consistent with these expectations in relations between work stress and depressive symptoms in other U.S. Latino samples of agricultural workers [8,10,17,25,26].

Family stress and ecocultural stress-based models [21,22], in turn, suggest that family relationship quality (e.g., conflict among family members) and positive youth outcomes might be undermined by primary caregivers who report high levels of stress, depressive symptoms, and lower levels of life satisfaction. These models posit that stress and injuries to primary caregivers can negatively impact family dynamics and youth prosocial behaviors (e.g., care-based actions that benefit others and reflect healthy social functioning; [22]). Specifically, one might expect greater family conflict episodes when a primary caregiver is experiencing difficulties resulting from injury or from high levels of stress at work. Such negative and challenging experiences can trigger strong negative arousal, deplete psychological resources, and dysregulate emotions, which can result in a proneness to more interpersonal conflict and tension (stress depletion and dysregulation hypotheses; [27]). Thus, we assert that work-related stress and injuries, which result in increased challenges and demands on caregivers, are also likely to deplete resources and undermine mental health, which can negatively affect family interactions and youth prosocial behaviors.

Although directly supportive evidence on work-related injuries and stress in caregivers and their relations to quality of family relationships and youth prosocial behavior is sparse, there is some supportive evidence on these expected relations in U.S. Latino families and youth. Prior work shows that stress experiences negatively impacted prosocial behaviors in youth [28,29,30,31]. One recent study also showed that stress is positively related to family conflict and negatively to youth prosocial behaviors [32]. Furthermore, Davis et al. [27] showed that discrimination events positively predicted U.S. Latino youth depressive symptoms in a longitudinal study, which in turn, negatively predicted prosocial behaviors. Moreover, in a meta-analytic review of the existing work, Memmott-Elison et al. [33] reported an overall negative relation between depressive symptoms and prosocial behaviors. However, the evidence on these expected relations is mostly limited to youth and family-related stress experiences (not work-related stress or discrimination) and youth prosocial behaviors.

### 1.3. Study Hypotheses

**Figure 1:**
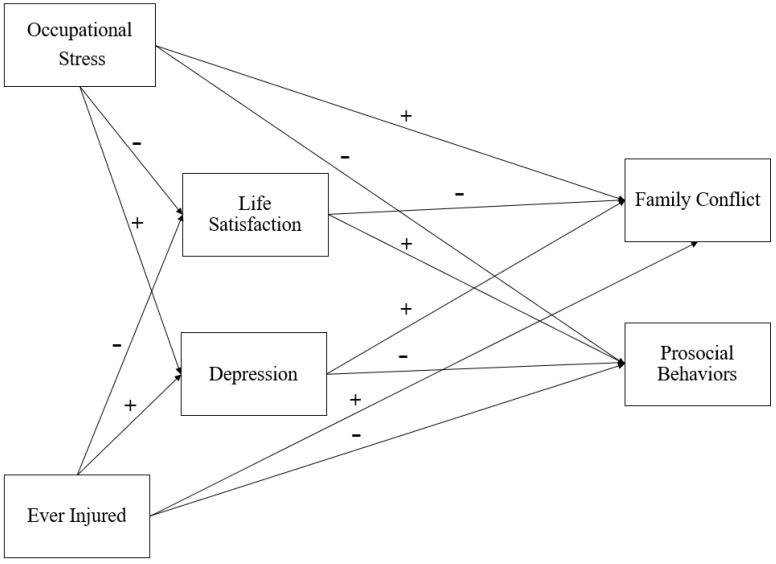
Conceptual model with expected relations among main study variables.

We examined the relations among occupational stress, injuries, family conflict, and youth prosocial behaviors. Moreover, we investigated whether workers’ life satisfaction and depressive symptoms mediated these relations. Based on primarily on ecocultural stress-based, family stress models and stress theories [20,21,22] and the limited prior research, we expected that occupation-related stress and injuries in cattle feedyard workers would be positively related to depressive symptoms, which in turn, would be positively linked to family conflict and negatively linked to youth prosocial behaviors. In contrast, we hypothesized that work-related stress and injuries would be negatively related to life satisfaction, and in turn, would be positively related to youth prosocial behaviors but negatively related to family conflict. We also hypothesized that work-related stress and injuries would be positively related to family conflict but negatively related to youth prosocial behaviors (see Figure 1).

## 2. Materials and Methods

### 2.1. Participants

To be eligible to participate in the study, workers had to be currently employed on a cattle feedyard in Kansas or Nebraska, be at least 18 years of age (the age of majority in the state where the interview was conducted), and identify as an immigrant of Hispanic/Latino descent. The current sample included 242 workers. Worker’s youth were mostly (79.2%) less than or equal to 19 years of age, including 58.4% that were between 10 and 19 years of age. Table 1 shows demographic characteristics of the current sample.

### 2.2. Procedures

The data was from the *Health and Safety Risks of Latino Immigrant Cattle Feedyard Workers in the Central States* project. The study was approved by the Institutional Review Board at the University of Nebraska Medical Center.

Face-to-face interviews with workers were conducted between May 2017 and February 2020 and were based on a structured questionnaire assessing physical health, occupational context, prevention opportunities, emotional health and stress, and demographics. Interviews were conducted with workers mainly outside of the worksite after working hours and could be completed in either English or Spanish based on the worker’s language preference. Twenty interviews were conducted at the jobsite during working hours. Workers received a $25 or $30 gift card (compensation was increased to $30 to enhance recruitment success for some participants) for participating in the study.

### 2.3. Measures

#### 2.3.1. Occupational Stress

Stress was assessed using nine items based on the Hispanic Stress Inventory (HSI), immigrant version [34]. Participants were asked if they have experienced each potential stressor within the last three months. If they responded “yes”, then they were asked how much stress the situation had caused them on a scale from *not at all stressful* (1) to *extremely stressful* (5). Sample items included, “Because I am Latino, I have been expected to work harder” and “I have had to watch the quality of my work so others do not think I am lazy”. A total score was obtained by summing the scores for each of the items, and higher scores indicated higher levels of stress. Inter-item correlations ranged from .34 to .69. The scale had good reliability in this sample; McDonald’s Ω = .89.

#### 2.3.2. Depression

The Center for Epidemiologic Studies Depression Scale (CESD-10) was used to assess depressive symptomology [35]. The scale consists of 10 items that assess how frequently a person reported symptoms associated with depression (e.g., sad, everything was an effort, or had restless sleep) over seven days. Participants could respond *rarely or none of the time* (*less than 1 day*) (*0*), *some or a little of the time* (*1–2 days*) (*1*), *occasionally or a moderate amount of time* (*3–4 days*) (*2*), *or all of the time* (*5–7 days*) (*3*). Items associated with a positive mood were reverse coded. A total score was calculated by taking the mean of all the items. The scale was not scored if two or more items were missing. A cutoff score equal to or above 10 was considered depressed. The scale demonstrated construct validity in prior research [36] and acceptable reliability in this sample; McDonald’s Ω = .74 with inter-item correlations (*r* = .02 to .52).

#### 2.3.3. Familial Conflict

In order to measure family conflict, items from the family conflict subscale of the Self-Report Family Inventory [37] were used in the current study. Ten items of this subscale were used to assess perceptions of conflict within the family. Response options were on a 5-point Likert scale ranging from *fits our household very well* (1) to *doesn’t fit our household at all* (5). Sample items included, “We argue a lot and never solve problems” and “When things go wrong, we blame each other.” Two items were worded in reverse. However, preliminary analyses indicated that these items lowered the internal reliability and were not used in the current study. The revised eight-item family conflict scale had acceptable reliability in this sample; McDonald’s Ω = .74 with inter-item correlations (*r* = .03 to .58).

#### 2.3.4. Prosocial Behaviors

Prosocial behaviors were measured using the Prosocial Tendencies Measure [38] (PTM). Workers reported on ten items that measured various forms of prosocial behaviors in their (oldest) youth, including emotional (sample item: “I usually help others when they are very upset”), dire (sample item: “I tend to help people who are in real crisis or need”), and compliant (sample item: “When people ask me to help, I don’t hesitate”) actions. Participants responded using a 5-point scale ranging from *does not describe me at all* (1) to *describes me greatly* (5). The scale showed construct validity in previous work [39] as well as acceptable reliability in this sample; McDonald’s Ω = .88 and interitem correlations (*r* = .23 to .69).

#### 2.3.5. Life Satisfaction

The Satisfaction With Life Scale (SWLS) was used to assess satisfaction with life as a whole [40]. The 5-item scale measures subjective well-being. Sample items included, “The conditions of my life are excellent and “So far I have gotten the important things I want in life.” Items were measured on a Likert-type scale from *strongly disagree* (1) to *strongly agree* (7). Total scores may range from 5 to 35, and there was acceptable internal consistency in this sample with McDonald’s Ω = .79 and inter-item correlations ranging from .32 to .56.

#### 2.3.6. Demographic Covariates

Participants were asked a series of demographic questions including age and gender (male or female). They were also asked to report on whether they had ever been injured working on a feedyard (yes or no).

### 2.4. Data Analysis Plan

Mplus version 8.0 [41] was used to examine a path model examining the direct and indirect relations among the main study variables. Two endogenous variables (family conflict, prosocial behaviors) were regressed onto depression and life satisfaction, as well as occupational stress and ever injured. Depression and life satisfaction were also regressed onto occupational stress and ever injured. We controlled for cattle feedyard workers’ age and gender on the mediating and endogenous variables in this model.

We requested the full information maximum likelihood (ML) estimator in order to estimate missing data, as well as (*N* = 5000) bootstraps. An indirect effect deemed to be significant if the 95% confidence interval for the estimate of the standard error estimate did not include zero that specific indirect effect [42]. As a fully saturated model (i.e., a model that provides exact fit of the data) was examined in the current study, we did not report any model fit indices.

## 3. Results

### 3.1. Descriptive Statistics and Correlations

Univariate and bivariate statistics for the main study and control variables can be found in Table 2. Occupational stress was negatively correlated with life satisfaction and positively correlated with depression. Those who reported a lifetime injury and men reported higher occupational stress. Additionally, those who reported a lifetime injury reported less life satisfaction, increased depression, were older, and were more likely to be men. Life satisfaction was inversely related to depression, but positively related to prosocial behaviors. Depression was positively related to family conflict and negatively correlated with prosocial behaviors. Prosocial behaviors were also positively correlated with age.

### 3.2. Path Analysis

**Figure 2:**
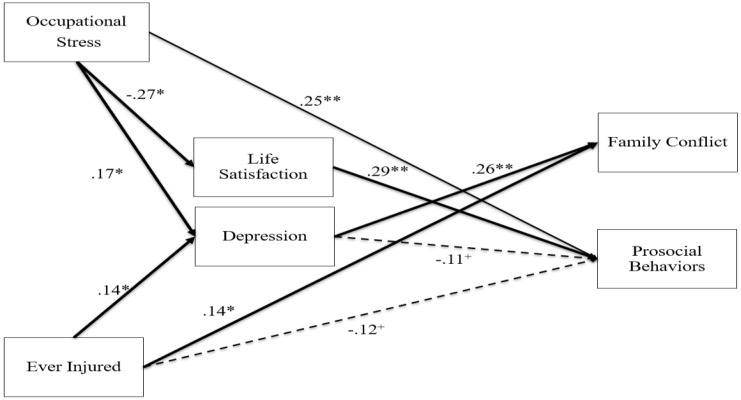
Occupational Stress and Lifetime Injury Predicting Life Satisfaction, Depression, Family Conflict, and Prosocial Behaviors. ^+^ *p* < .10, * *p* < .05, ** *p* < .01. Note: The 95% C.I. for all bootstrapped indirect effects, indicated by bolded lines, fell outside of zero. Solid lines represent direct effects, and dashed lines represent paths that approached statistical significance. Nonsignificant paths, the significant covariances among the endogenous variables, and paths including control variables have been omitted from the figure for parsimony.

Results for the path model can be found in Figure 2. Three indirect paths were found in the current model. The 95% confidence interval for the unstandardized indirect effect involving occupational stress, life satisfaction, and prosocial behaviors fell outside of zero (−.09, −.02). Additionally, the 95% confidence interval for the unstandardized indirect effect involving occupational stress, depression, and family conflict fell outside of zero (.00, .05). Finally, the 95% confidence interval for the unstandardized indirect effect involving lifetime injury, depression, and family conflict fell outside of zero (.00, .10). In addition to the indirect effects, two direct effects were significant. Higher levels of occupational stress were related to greater prosocial behaviors, and lifetime injury was related to greater family conflict.

## 4. Discussion

The current findings suggest that increased occupational stress and injuries on cattle feedyards predict internalizing symptomatology, and in turn, internalizing symptomatology is linked to heightened conflict at home. Additionally, occupational stress was linked to lower life satisfaction, which in turn, was related to engaging in fewer prosocial behaviors. The findings yield evidence that injuries and stress in the workplace can spill over to the family context and undermine health, family dynamics, and youth wellbeing. These findings were robust across age and gender and are in accord with the ecocultural stress-based model that delineates how stressors and injuries in the workplace can impact psychological and behavioral adjustment [19].

Of particular interest were the findings demonstrating indirect links between occupational stress and injuries to heightened family conflict via increased levels of depressive symptoms. These findings suggest that family functioning could be impacted by physical (i.e., injuries) and psychological (i.e., felt stress) factors that occur in the workplace. Psychological functioning (i.e., depressive symptoms) can be undermined when persons experience relatively high levels of stress and physical health problems. Stress theorists, for example, note that persons’ psychological resources can be depleted under such circumstances, which can impact attentional processes, induce negative emotionality, and reduce persistence. These consequences, in turn, could facilitate negative interactions among family members and lead to conflict and tension. Because family members can also experience stress, future research is needed to discern whether family stress can also have reciprocal effects on workers’ stress and proneness to work-related injuries.

As expected, there was also an indirect relation between occupational stress and youth prosocial behaviors via workers’ life satisfaction. Specifically, higher levels of work-related stress were associated with lower worker-reported life satisfaction, which in turn, was related to lower levels of youth prosocial behaviors. This pattern of relations revealed that work-related stress was not only linked to family functioning but can also have effects on youth socioemotional development. Researchers have shown that warm, nurturant parent-child relationships are positively related to youth prosocial behaviors [43]. Perhaps workers who report relatively low levels of life satisfaction due to exposure to work-related stress might also manifest poorer quality relationships with their youth resulting in relatively low levels of youth prosocial behaviors. To our knowledge, these are first empirical findings demonstrating links between caregivers’ work-related factors and their youth prosocial behaviors, and as such this work extends prior theories of youth prosocial development that focus on home-related factors and youth characteristics.

Experiencing an injury was also directly related to fewer prosocial behaviors, suggesting that workplace safety may jeopardize positive social interactions for reasons beyond mental health symptomatology or life satisfaction. Conversely, occupational stress was directly and positively related to prosocial behaviors. Prior research has similarly found that acculturative stress may orient Latinos to the emotional needs of others, which in turn promotes helping behaviors [44]. This notion, referred to as altruism born of suffering, is theorized to account for why some persons who are exposed to adversity overcome such obstacles and develop a strong prosocial orientation [45]. Furthermore, there is evidence that persons sometimes help others when under distress to improve their own mood [46]. This finding, thus, requires more research. However, we note that family conflict was not directly predicted by workplace stressors, suggesting that negative social outcomes may be particularly attributed to the indirect carry-over effects of workplace stress.

The findings have several important applied implications. Interventions that support farmworkers and their families in managing stress and mitigating the negative impact of injuries on both worker and family wellbeing are needed. Mental and behavioral healthcare providers are encouraged to work in tandem with community organizations to disseminate information and resources on stress and mental health. Such partnerships may facilitate trust with the farmworker community and promote the use of culturally, linguistically, and contextually appropriate strategies for outreach, health education, and engagement. Furthermore, the findings from this study, including the impact of stress and injuries on mental health, life satisfaction, family conflict, and youth prosocial behaviors, should be integrated into structural competency training initiatives for healthcare providers and agricultural employers [47].

Preventing workplace injuries and reducing their work-related stress is critical. Unfortunately, occupational injuries in agriculture are common, even though they are usually underreported [2,48]. Developing a culture of safety, one that prioritizes worker wellbeing, can be an important preventive strategy to reduce the risk of occupational injuries and stress. Such efforts could include building a foundation based on the principles of relationship-centered leadership (e.g., communication, trust, and care) described by Carrillo (2020) [49], which could be especially important for Latino immigrant workers who may identify closely with cultural values such as *personalismo* (i.e., warm, caring, and trusting relationships with others) [50,51]. Efforts may also highlight management’s commitment to safety by ensuring that safety is a visible priority of the operation, providing regular safety training and updates to workers, and ensuring that safety equipment is consistently available and accessible. Feedyard operations are encouraged to find ways to engage workers in safety initiatives so that they are active participants in creating a culture of safety, rather than just passive recipients. Safety should be more than compliance [52].

Although prevention efforts are an ideal as a mechanism to reduce stress and the likelihood of injuries, access to more healthcare and supportive services might also be warranted. For example, ensuring more farmworkers have access to workers’ compensation coverage could be beneficial to injured workers [53] and ease the potential negative impact on family relationships and their youth development. Increasing access to mental health and other healthcare services through distance health technologies (i.e., telehealth, mHealth) in rural communities where many cattle feedyards exist, funding programs to incentivize more healthcare workers to rural practice, and supporting more health outreach programs might be viable options. Another opportunity might be for feedyard employers to partner with healthcare institutions to provide onsite health services for feeedyard workers and/or their families on a regular basis or to develop incentive programs such as tax subsidies or discounts on health insurance premiums for preventive health services, thereby normalizing preventive healthcare and reducing stigma for help-seeking. Alternatively, cattle feedyard workers and their families might be able to access support through nearby community centers or schools.

The present findings should be interpreted with caution given study limitations. First, the measures relied on workers’ reports. Because reliance on one reporter can produce biases, future studies using multiple reporters (e.g., youth reports) and behavioral or biological markers would be desirable. Second, the study was a cross-sectional design, which limits the ability to make strong inferences regarding causality and direction of effects such as the possibility of bidirectional effects between family conflict and depression and life satisfaction. For example, as noted previously, it is possible that family conflict influences workers’ safety and injuries. Longitudinal and intervention designs would strengthen our ability to make stronger causal inferences. Third, future research would benefit from examining how the findings might differ across different types of injuries. It might be the case that effects for injury might be stronger when injuries are more severe. Finally, a larger and more representative study sample (e.g., other parts of the U.S., multiple Latino subgroups) is needed to examine the generalizability of the present findings.

## 5. Conclusions

Despite these limitations, the present findings inform existing models of immigrant cattle feedyard workers’ health and family wellbeing. Occupational stress and lifetime injuries were predictive of workers’ psychological adjustment, which in turn, predicted family and youth behavioral outcomes. Moreover, direct predictive paths yielded additional evidence on the effects of occupational stress and work-related injuries on family and youth wellbeing. These findings are the first to demonstrate the deleterious possible consequences of work-related stress and injuries in U.S. Latino immigrant cattle feedyard workers and their families and youth. Such findings suggest the need for greater attention to stress and injuries in the workplace and their possible impact on family functioning and point to the development of intervention efforts aimed at reducing such stress, increasing access to mental health services, and developing a strong safety culture, including a robust safety training program.

## Figures and Tables

**Table 1:** Demographic characteristics of the sample (*N* = 242).

Demographic Characteristics	Descriptive Statistics
Age in years—Mean (SD)	
Worker’s age Mean (SD)	37.72 (10.11)
Youths’ age Mean (SD)	15.03 (7.31)
Gender (%)	
Male	90.9%
Female	9.1%
Country of origin (%)	
Mexico	69.5%
Guatemala	17.3%
El Salvador	6.2%
Honduras	2.5%
Cuba	2.5%
Other countries	2%
Years in the U.S.—Mean (SD)	12.23 (9.64)

**Table 2:** Bivariate correlations and descriptive statistics for main study and control variables.

	1	2	3	4	5	6	7	8
(1) Occupational Stress	--							
(2) Ever Injured ^a^	.23 **	--						
(3) Life Satisfaction	−.30 **	−.17 **	--					
(4) Depression	018 *	.13 **	−.37 **	--				
(5) Family Conflict	.13 *	.20 **	−.11	.28 **	--			
(6) Prosocial Behaviors	.11 ^+^	−.11^+^	.29 **	−.19 **	−.08	--		
(7) Age	−.01	.16 *	.07	−.04	.12 ^+^	.14 *	--	--
(8) Gender ^b^	−.14 **	−.31 **	.11 ^+^	.05	−.04	.04	.01	--
Mean	.82	.72	5.44	.39	1.40	4.19	37.72	.09
Standard Deviation	1.08	--	.47	.38	.54	.68	10.08	
*N*	242	242	242	240	238	241	242	242

^+^ *p* < .10, * *p* < .05, ** *p* < .01. ^a^ Ever Injured coded as 0 = Never injured, 1 = Injured at least once. ^b^ Gender coded as 0 = Male, 1 = Female. Note: Standard Deviations were not calculated for dichotomous variables.

## Data Availability

The data presented in this study are available on request from Athena Ramos. The data are not publicly available due to privacy restrictions.
